# Do Aspects of Protein Intake Vary Across the Week in Healthy Community-Dwelling Older Adults?—An *enable* Study

**DOI:** 10.3390/nu10091217

**Published:** 2018-09-03

**Authors:** Anne Gingrich, Rachel Rennekamp, Beate Brandl, Thomas Skurk, Hans Hauner, Cornel C. Sieber, Dorothee Volkert, Eva Kiesswetter

**Affiliations:** 1 Institute for Biomedicine of Aging, Friedrich-Alexander-Universität Erlangen-Nürnberg, Kobergerstraße 60, 90408 Nürnberg, Germany; cornel.sieber@fau.de (C.C.S.); dorothee.volkert@fau.de (D.V.); eva.kiesswetter@fau.de (E.K.); 2 Chair of Nutritional Medicine, Technical University of Munich, Gregor-Mendel-Str. 2, 85354 Freising, Germany; rachel.rennekamp@tum.de (R.R.); beate.brandl@tum.de (B.B.); skurk@tum.de (T.S.); hans.hauner@tum.de (H.H.); 3 ZIEL Institute for Food and Health, Core Facility Human Studies, Technical University of Munich, Gregor-Mendel-Str. 2, 85354 Freising, Germany; 4 Institute of Nutritional Medicine, Klinikum rechts der Isar, Technical University of Munich, Georg-Brauchle-Ring 62, 80992 Munich, Germany; 5 Krankenhaus Barmherzige Brüder Regensburg, Prüfeninger Straße 86, 93049 Regensburg, Germany

**Keywords:** weekend-weekday, protein intake, protein distribution, protein source, aging

## Abstract

Various aspects of protein intake are thought to be crucial for the prevention of sarcopenia in older adults. Information about the day-to-day variation in these aspects is lacking. Our objective was to examine whether daily protein intake, protein distribution across meals, number of meals providing adequate protein, and protein sources vary across the week in healthy community-dwelling older adults. In 140 persons (51% women) that were aged 75–85 years, protein intake was assessed by seven-day food records. On average across the week, protein intake (median [IQR]) was 0.93 [0.79–1.10] g/kg body weight (BW) and the coefficient of variation across the three main meals was 0.50 [0.40–0.61]. The number of meals per day providing ≥0.4 g protein/kg BW was 0.57 [0.43–1.00] and 60.0 [52.4–65.2]% of protein intake was animal-based. According to Friedman’s test, differences throughout the week were observed in women for daily protein intake (*p* = 0.038; Sunday: 0.99 [0.78–1.31] vs. Tuesday: 0.79 [0.68–1.12] g/kg BW) and number of meals with adequate protein (*p* = 0.019; ≥1 daily meal: Sunday: 69.4% vs. Tuesday: 41.7%). On Sunday, protein intake was most in agreement with suggestions to prevent sarcopenia. In men, protein intake did not differ throughout the week.

## 1. Introduction

For the maintenance of muscle mass in older persons and the prevention of sarcopenia, several aspects of protein intake are suggested to be relevant. These are a daily intake of at least 1.0 g/kg BW (body weight), an even distribution of the daily amount across the main meals, a per-meal amount of at least 0.4 g/kg BW, and a high protein quality, i.e., a high amount of animal-based protein [1,2,3,4,5,6]. In order to prevent or delay the onset of sarcopenia and to regularly stimulate muscle protein synthesis and inhibit breakdown, experts recommend meeting these aspects of protein intake every day of the week and at every meal [3].

There is evidence primarily in middle-aged adults that dietary intake differs throughout the week. On the weekends, more energy, fat, carbohydrates, and alcohol are consumed [7,8,9], and meal sizes are larger [8]. These differences are attributed to social, psychological, and environmental factors [8,10]. A few studies in older adults showed that protein [7] or fat intake [8,11] were higher on the weekend compared to weekdays, but these weekend-weekday differences were less pronounced than in younger individuals. Overall, little is known about the variation of protein intake from day to day in older adults and detailed information on the adherence to suggested aspects of protein intake across the week is lacking. Traditional eating habits, like eating fish on Fridays or a Sunday roast, as well as eating in a restaurant or together with relatives or friends on the weekend might affect the amount and quality of protein intake. While excessive energy intake on the weekend might play a negative role in long-term weight management in younger adults [10,12], in older adults, a greater dietary intake and particularly protein supply might be beneficial for the maintenance of muscle mass.

In several previous studies showing an association between protein intake and muscle mass and function, protein intake was assessed by 24-h dietary recalls or food records on a maximum of three days [13,14,15,16,17,18,19,20,21]. These studies used the mean protein intake for the analysis and did not take potential variations in protein intake across the week into account. Information on the day-to-day variation in the aspects of protein intake would be valuable for the adequate assessment of protein intake. The identification of specific days of the week with more favorable protein intake would be useful for dietary counselling with respect to the prevention of sarcopenia. However, to the best of our knowledge, the variation in the above-mentioned aspects of protein intake across the week has not been investigated in older adults yet. Thus, it was our objective to examine whether the daily amount of protein intake, the distribution of protein across the main meals, the number of meals providing an adequate amount of protein, and the protein sources differ between the seven days of the week in healthy, community-dwelling older adults.

## 2. Materials and Methods

### 2.1. Study Design and Participants

For this cross-sectional, multi-center study, community-dwelling healthy Caucasian adults aged 75–85 years were recruited between April 2016 and March 2018 in Nuremberg and Freising, Germany. We aimed at recruiting 160 persons, 100 persons from Nuremberg and 60 from Freising, with equal proportions of men and women. Exclusion criteria were: BMI less than 18.5 or greater than 35 kg/m², smoking, immobility, need of care, unintended weight loss of more than 5% in the previous three months, cognitive impairment (Mini Mental State Examination (MMSE) <24 points), blood transfusion in the previous three months, and current participation in intervention studies. In addition, the following physician-diagnosed chronic diseases (self-reported, medication list) led to exclusion: human immunodeficiency virus infection, liver disease, diabetes mellitus, endocrine disease, autoimmune disease, renal failure requiring dialysis, lung disease, stomach ulcer, or the occurrence of heart failure, stroke, coronary heart disease, untreated hypertension, cancer, and psychological and neurological or neurodegenerative diseases within the previous three years.

The study was conducted according to the guidelines that were laid down in the Declaration of Helsinki and all the procedures were approved by the ethics committees of the Friedrich-Alexander-Universität Erlangen-Nürnberg (number: 291_15 B) and the Technical University of Munich (number: 452/15). Written informed consent was obtained from all participants prior to inclusion. The study was registered at the German Clinical Trials Register (DRKS-ID: DRKS00009797).

General study eligibility was screened by a systematic telephone interview, and individuals meeting the inclusion criteria were invited to the study centers for a screening. If eligible, they were scheduled for two test days, each approximately 5 h in length. Participants were asked to refrain from any intense physical activity the evening before and the morning of the two test days. More detailed information on study procedures is published elsewhere [22].

### 2.2. Assessment of Paticipants’ Characteristics

Information on participants’ living situation (alone/with partner/with children/with grandchildren/with relatives of same generation/with others), eating companion (alone/with partner/with relatives/with others), and appetite (very good/good/medium/poor/very poor) was obtained by standardized self-report questionnaires.

Depressive symptoms were screened using the Geriatric Depression Scale (GDS; 0–15 points) [23]. A score of >7 points indicates a depressed mood [24].

The cognitive status was rated with the MMSE (0–30 points) [25]. Higher scores indicate better cognitive status.

The nutritional status was evaluated with the Mini Nutritional Assessment (MNA; 0–30 points) [26]. A score ≥24 indicates a normal nutritional status, between 23.5 and 17 indicates a risk of malnutrition and a score <17 indicates malnutrition.

The Short Physical performance Battery (SPPB; 0–12 points), including tests for balance (side-by-side, semi-tandem, tandem stances), usual gait speed (4 m course), and functional lower extremity strength (sit-to-stand, five repetitions) was applied to assess physical functional status [27]. Each domain scores 0–4 points, with higher scores indicating better performance. An overall sum score was calculated [27].

Body height (in cm, to the nearest 0.1 cm) was measured without shoes while using a stadiometer (Seca, Hamburg, Germany). BW (in kg, to the nearest 0.05 kg) and body composition, including fat free mass in kg by Bioelectrical Impedance Analysis (BIA), were assessed using a Seca medical Body Composition Analyzer 515 (Seca, Hamburg, Germany) after overnight fasting. At the study site in Freising, participants were weighed in underwear. In Nuremberg, the participants wore light clothes and no shoes, and thus weight was corrected by subtracting 1 kg. Skeletal muscle mass (SMM) was calculated according to the equations of Janssen et al. [28] and then divided by height squared to obtain the skeletal muscle index (SMI, kg/m²).

### 2.3. Aspects of Protein Intake

Dietary intake was assessed while using an open seven-day food record. At the screening visit, the participants were provided with detailed written and oral instructions by nutritional scientists. All foods and beverages, portion sizes (using a scale or household measures), and the time of intake were reported. Participants were instructed to stick to their usual dietary habits. The last reporting day was the day prior to the first test day. On test day 1, the records were checked for completeness and, if necessary, participants were asked for additional information.

Food intake was categorized into ‘breakfast’, ‘morning snack’, ‘lunch’, ‘afternoon snack’, ‘dinner’, and ‘evening snack’ based on time of intake and size/composition of meal. Energy and macronutrient intake were calculated per day and per meal using EBISpro software (EBISpro, Willstätt-Legelshurst, Germany, 2016) based on the German nutrient database ‘Bundeslebensmittelschlüssel’ (version 3.02, Karlsruhe, Germany) [29]. Data entry was checked by a second nutritional scientist.

The evenness of protein distribution across the three main meals was expressed by the dimensionless coefficient of variation (CV) of protein intake at breakfast, lunch, and dinner (CV = standard deviation/mean)—the smaller the CV, the more even the distribution. Furthermore, the intra-individual variation in daily protein intake across the week was assessed based on the CV of the daily protein intake.

Protein intake per day and per meal in g was divided by participants’ BW in kg to obtain the relative intake. The number of meals per day containing ≥0.4 g protein/kg BW was counted with a potential maximum number of six meals.

For every reported food item, the amount of protein (g) was assigned to one of eight protein source categories: Three plant-based protein sources, namely ‘starchy foods’, ‘fruits, vegetables, pulses, nuts and seeds’ and ‘other predominantly plant-based protein sources’ and five animal-based protein sources namely ‘meat and meat products’, ‘dairy products’, ‘eggs and egg products’, ‘fish and seafood’, and ‘other predominantly animal-based protein sources’ were considered. In the case of mixed foods, the component(s) with the highest protein content led to categorization based on common recipes.

### 2.4. Data Analysis and Statistics

Statistical analyses were performed with SPSS Version 24 (IBM SPSS Statistics, Chicago, IL, USA). Data were tested for normality for men and women separately using the Shapiro-Wilk test and they are presented as mean ± standard deviation (SD) or median and interquartile range (IQR). Differences in continuous characteristics between men and women were tested using Mann-Whitney U test or independent samples *t*-test (normally distributed). For categorical characteristics, Chi² test was applied. In order to determine whether aspects of protein intake differ between the seven days of the week in men or women, Friedman’s test (dependent samples) was used. In the case of significant difference, pairwise Dunn-Bonferroni Post-hoc test adjusted by Bonferroni correction for multiple tests was applied to locate these differences. The level of significance was set at *p* < 0.05.

## 3. Results

### 3.1. Characteristics of the Study Participants

Three hundred and sixty-nine individuals were screened for eligibility by telephone calls. Two hundred and nine did not fulfill all the inclusion criteria. Of the 160 eligible and tested individuals, 19 participants were excluded from the present analysis because of a dietary recording period of less than seven days and one man was excluded because of a mean energy intake of more than three SD from the mean. In total, 140 participants (104 from Nuremberg; 36 from Freising), hereof 72 women, were included in the present analyses.

Participants’ characteristics are presented in Table 1. Mean age (±SD) was 78.2 ± 2.8 years and the mean BMI was 26.6 ± 4.0 kg/m². Nutritional status was normal in 94.3% of participants according to MNA and no participant had malnutrition. The functional status was good in 95.0% according to SPPB (>10 points). None of the participants rated his/her appetite as poor or very poor and 12% reported moderate appetite. More than half of the participants reported living alone and eating their main meal usually alone. These proportions were higher in women than in men (Table 1).

The mean energy intake was 1733 ± 330 kcal/day in women and 2121 ± 444 kcal/day in men. The mean protein intake was 63.9 ± 14.6 g/day in women and 78.1 ± 20.3 g/day in men (Table 1).

### 3.2. Daily Protein Intake Across the Week

Median [IQR] daily protein intake on average across the week was 0.93 [0.79–1.10] g/kg BW and 15.3 [13.3–17.0]% of energy intake without differences between men and women (*p* = 0.793 and *p* = 0.720, respectively).

Intra-individual variation in daily protein intake, as measured as CV of protein intake across the seven weekdays, was 0.24 [0.19–0.29] (minimum: 0.07; maximum: 0.53) and it was also not different between men and women (*p* = 0.636).

**Figure 1:**
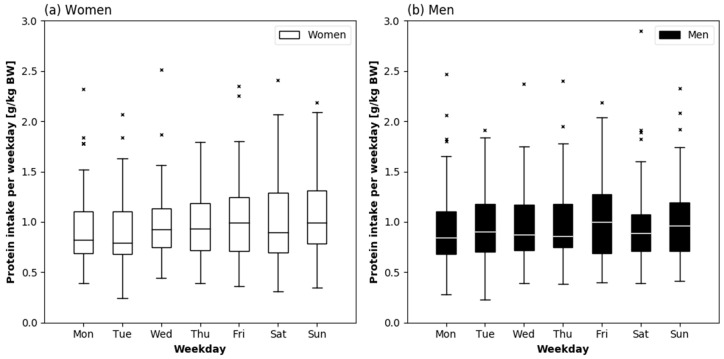
Daily protein intake relative to body weight (BW) by weekday in healthy community-dwelling older women (*n* = 72) (**a**) and men (*n* = 68) (**b**). Friedman’s test: *p* = 0.013 in women, *p* = 0.198 in men. The boxes represent interquartile ranges with the horizontal lines denoting the median. The whiskers show the highest and lowest values within the 1.5-fold interquartile range. The × represent outliers.

**Figure 2:**
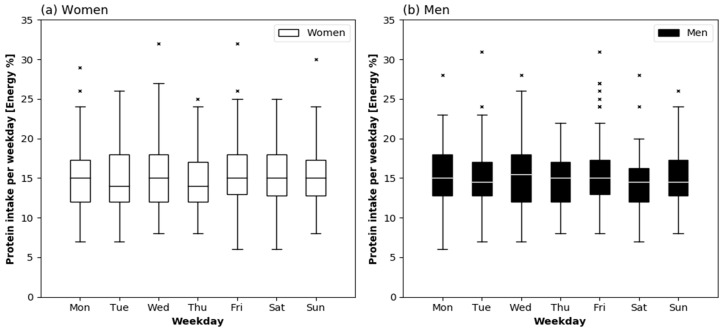
Daily Protein intake in percent of total energy intake by weekday in healthy community-dwelling older women (*n* = 72) (**a**) and men (*n* = 68) (**b**). Friedman’s test: *p* = 0.705 in women, *p* = 0.557 in men. The boxes represent interquartile ranges with the horizontal lines denoting the median. The whiskers show the highest and lowest values within the 1.5-fold interquartile range. The × represent outliers.

Protein intake relative to BW and in percent of energy intake on each day of the week are presented stratified by sex in Figure 1 and Figure 2, respectively. In women, protein intake in g/kg BW was different across the week (*p* = 0.038), and according to the Post-hoc test higher on Sunday (0.99 [0.78–1.31] g/kg) as compared to Tuesday (0.79 [0.68–1.12] g/kg) (*p* = 0.013). Protein intake in percent of energy intake was, however, not different across the week in women (*p* = 0.705). In men, both variables did not differ across the week.

### 3.3. Distribution of Protein Intake between the Three Main Meals across the Week

On each of the seven weekdays, the median number of daily meals was 4.0 [4.0–5.0] with no difference across the week, in either in women or in men. The median CV across the three main meals on average over the seven days was 0.50 [0.40–0.61] without any difference between men and women (*p* = 0.245).

**Figure 3:**
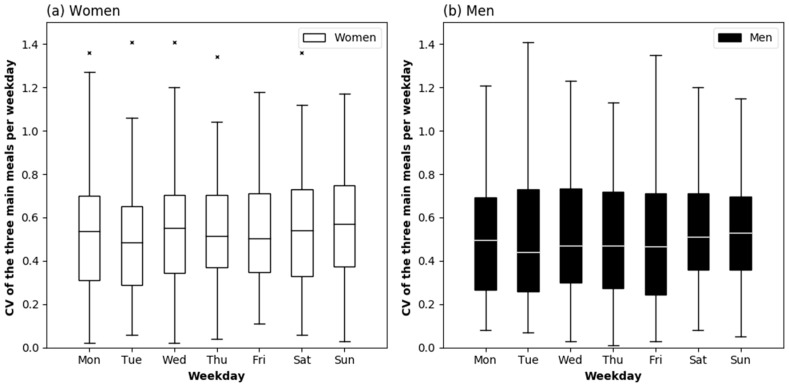
Protein distribution across the day expressed as coefficient of variation (CV) between three main meals by weekday in healthy community-dwelling older women (*n* = 72) (**a**) and men (*n* = 68) (**b**). Friedman’s test: *p* = 0.350 in women, *p* = 0.147 in men. The boxes represent interquartile ranges with the horizontal lines denoting the median. The whiskers show the highest and lowest values within the 1.5-fold interquartile range. The × represent outliers.

Figure 3 shows the distribution of protein intake across the three main meals as CV for each weekday, separately for men and women. No differences were observed across the week in women or men.

### 3.4. Number of Meals Providing Adequate Protein across the Week

The daily number of meals providing at least 0.4 g protein/kg BW on average across the week was 0.57 [0.43–1.00], with no difference between men and women (*p* = 0.563). Using the Friedman’s test, the daily number of meals with adequate protein differed across the week in women (*p* = 0.019). Pairwise post-hoc test adjusted by Bonferroni correction for multiple tests did not, however, identify that certain days that were different. In men, no difference in the daily number of meals with adequate protein intake was observed across the week (Friedman’s test *p* = 0.536).

**Figure 4:**
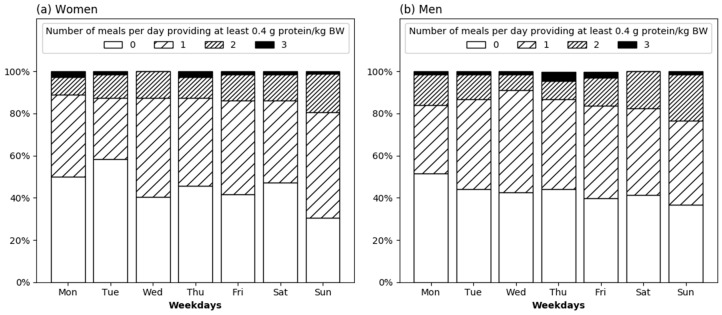
Proportion of healthy community-dwelling older women (*n* = 72) (**a**) and men (*n* = 68) (**b**) consuming 0, 1, 2 or 3 daily meals providing at least 0.4 g protein/kg body weight (BW) by weekday.

The proportion of men and women consuming 0, 1, 2, or 3 daily meals providing at least 0.4 g protein/kg BW by weekday is displayed in Figure 4. None of the participants consumed more than three meals per day providing this amount of protein. On Sunday, the highest proportion (69.4%) and on Tuesday, the lowest proportion (41.7%) of women met the recommendation of 0.4 g protein/kg BW per meal at least once a day. Two or more meals were also most often reached on Sunday (19.4% of women). In men, the rate of participants consuming at least one daily meal with adequate protein was also highest on Sunday (63.2%) and lowest on Monday (48.5%). The rate of men consuming two or more meals with adequate protein was, as well, highest on Sunday (23.5%).

### 3.5. Protein Sources across the Week

**Figure 5:**
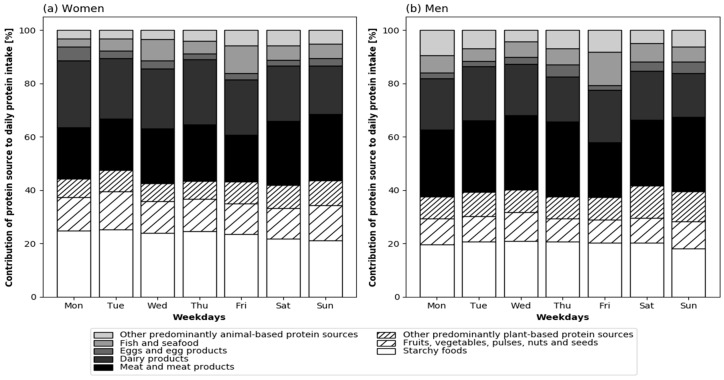
Contribution of protein sources to protein intake by weekday in healthy community-dwelling older women (*n* = 72) and men (*n* = 68). Mean is presented. Friedman’s test: egg and egg products: *p* = 0.033 in women, *p* = 0.012 in men, fish and seafood: *p* = 0.004 in women. For all other protein sources *p* ≥ 0.05.

The median [IQR] proportion of animal protein on average across the week was 58.6 [50.3–63.5]% of the total protein intake in women and 61.3 [53.8–66.9]% in men (*p* = 0.021). The contribution of the main protein sources to total daily protein intake across the seven weekdays is displayed in Figure 5, separately for men and women. The proportion of animal protein was not different across the seven weekdays in women (*p* = 0.459) or in men (*p* = 0.264). When looking at particular protein sources, the contribution of eggs and egg products to total the protein intake was different between the seven weekdays in women (*p* = 0.033) and in men (*p* = 0.012); however, the pairwise Post-hoc test did not reveal significant pairs. Furthermore, the proportional intake of protein deriving from fish and seafood was different across the week in women (*p* = 0.004). The proportion of fish and seafood intake was highest on Friday in both sexes (Figure 5), but the pairwise Post-hoc test did not identify typical fish and seafood days. No other differences in the contribution of protein sources were observed across the week.

## 4. Discussion

To the best of our knowledge, this is the first study describing the variation of various aspects of protein intake across the week in healthy community-dwelling older adults. Overall, the variation of these aspects was rather moderate across the week and a wide inter-individual variation was observed. In women, differences between the seven weekdays were found for the total daily protein intake (g/kg BW), number of meals with adequate amount of protein, as well as protein intake from eggs and egg products and fish and seafood. Sunday appears to be the day that is most in agreement with the suggestions for the maintenance of muscle mass, however, not in line with the per-meal concept of three daily meals with at least 0.4 g/kg BW [4].

Protein intake was assessed by seven-day food records. This approach is time-intense for participants as well as study staff. However, only this method allows the analysis of intra-individual variation of dietary intake over a full week. Only one previous study in older adults assessed food intake over a seven-day period, but clustered Monday through Thursday (weekdays) vs. Friday through Sunday (weekend days) [8]. In our sample, this categorization of raw data did not seem reasonable, since protein intake on Sunday was special. Other studies with larger sample sizes assessed only two independent days per participant and pooled weekdays and weekend days across different participants [7,11].

As intended, our participants were healthy and in a good emotional, cognitive, nutritional, and functional status. Daily protein intake was similar or slightly less than previously reported in healthy older persons of the same age [13,17,21,30,31], and slightly below the recommendation of 1.0 g/kg BW for older adults of the Germany Society for Nutrition [32]. In line with previous results in older adults (mean age 78 years) in the United Kingdom (UK), the suggested per-meal threshold of 0.4 g protein/kg BW was only rarely reached [33]. In the present study, protein intake was more evenly distributed across the main meals than in the UK (CV: 0.67 ± 0.2) [33] and another study with non-frail adults with a median age of 82 years from Germany (CV: 0.68 [0.15–1.24]) [30]. However, Figure 3 shows that the inter-individual variation of the CV was large on each day of the week. The proportion of animal protein per day (Figure 5) was slightly above a representative value for Europe (57%) that was calculated by Gorissen and Witard [6], based on aggregated representative data by the Statistics Division of FAO. This indicates that protein quality was generally good.

Up to now, the number of studies on the variation of dietary intake across the week in older adults has been very limited [7,8,11] and one of these studies did not report protein intake [11]. In 46 American adults aged ≥65 years, no difference in protein intake (percent of energy intake) between Monday through Thursday (weekdays) vs. Friday through Sunday (weekend days) was observed [8]. In 2530 adults aged ≥70 years from the United States (US), a 0.3% higher energy intake from protein was observed on a weekend day compared to a weekday [7]. Interestingly, in the age groups younger than 70 years in this study, the percentage of protein of total energy intake was lower on weekend days as compared to weekdays [7]. While we did not find a difference across the week for protein intake expressed as percent of total energy intake, the protein intake relative to BW was in women. In terms of muscle metabolism and compliance to dietary recommendations [2,32], the protein intake relative to BW is more relevant than protein intake in percent of energy intake but has not been investigated before. Moreover, the present work expands these general observations on proportion of macronutrient intake by a detailed characterization of aspects of protein intake across the week.

Previous studies have shown that week-weekend differences in dietary intake might be explained by social and environmental factors [8,10], and that older adults are responsive to these influences [8]. In our study, women lived more often alone and ate their main meals more often alone than men (Table 1). Women also had a ‘better’ protein intake on Sunday (than on Tuesday), which might be explained by eating with company and/or more traditional eating habits on Sundays. This speculation needs to be confirmed in future studies. In regard to dietary counselling, the better protein intake on Sunday is an interesting observation since older women could be encouraged to eat more often like they do on Sundays.

As indicated by the CV of daily protein intake across the week, some individuals consume their protein in a very uniform manner throughout the seven weekdays (minimum CV: 0.07), while others show a more varying protein intake (maximum CV: 0.53). Future research needs to investigate which protein intake pattern across the week is more beneficial regarding muscle mass preservation.

Our results show that the threshold of 0.4 g protein/kg BW is difficult to reach and only a few participants consumed such an amount of dietary protein three times daily (Figure 4). The proportion of older adults eating at least one or two meals with this amount of protein was highest on Sunday. The question may arise, whether a few weekly meals might be sufficient to support the maintenance of muscle mass, as habituation to regular high per-meal protein intake might occur. There is one experimental study in older men investigating a 14-day habituation to low (0.7 g protein/kg BW/day) vs. high protein intake (1.5 g protein/kg BW/day) that showed no subsequent changes to postprandial muscle protein synthesis rates after ingestion of 25 g protein [34]. This observation provides evidence that protein anabolism is not diminished when every meal contains sufficient protein but is also not augmented when just a few meals per week provide adequate protein. From a theoretical point of view, this means the more meals above the threshold, the better for the muscle. However, the present study demonstrates that the per-meal concept [4] is not applied under real-life conditions. Whether there is a long-term benefit of substantial modification of the diet towards three meals per day with a protein content above 0.4 g/kg BW [4] needs to be evaluated in future studies.

The proportion of animal protein intake in our study was stable across the week in both sexes (Figure 5). The intake of egg protein varied across the week, but no specific day(s) high or low in egg protein were identified. Overall, the contribution of the egg protein to protein intake was small since dietary guidelines restricted the intake off egg protein for more than half a century [35], and thus, not many people consume eggs daily. Fish and seafood was the other protein source that revealed differences across the week, though only in women and not in men. Nevertheless, Figure 5 points towards a higher proportion of fish and seafood protein on Friday than on the other days of the week. Thereby, Friday as traditional fish day may encourage particularly older people to eat fish on a regular basis. Fatty fish, possibly due to the additional n-3 fatty acids, was shown to be associated with grip strength [36].

A major strength of the present study is the detailed investigation of several aspects of protein intake across the week. Dietary assessment over a seven-day period in each participant is more comprehensive and detailed than in previous studies on weekday-weekend comparisons [7,11]. Thirdly, the categorization of protein sources was in line with previous studies [37,38] to ensure comparability of our results with published data.

One limitation is the regional focus of the study. One could speculate that especially the contribution of protein sources to protein intake might differ across regions and countries. Despite intensive employee training and the use of standard operating procedures, a centre bias cannot be excluded. Dietary intake was prospectively self-reported. In order to minimize the risk of misreporting, the participants were instructed to stick to their usual diet, every protocol was thoroughly checked for completeness, and missing information was immediately collected. Furthermore, it was previously shown that the underreporting rate is lower for protein intake than for energy intake [39]. To avoid an effect of the season on dietary intake, recruitment was spread over the course of one year with similar proportions of participants in each season. Moreover, no difference in protein intake (g/kg BW/day) between persons, who participated in the summer term (49.3% of participants) and winter term was observed (*p* = 0.700, data not shown).

## 5. Conclusions

In summary, the present study showed that the investigated aspects of protein intake are rather stable across the seven weekdays in community-dwelling healthy older men and women. Nevertheless, a small variation in protein intake across the week was observed, particularly in women. Overall, the adherence to the suggestions of protein intake for the maintenance of muscle mass was rather low, but on Sunday, protein intake was most in agreement with these recommendations. Future studies need to evaluate whether there is a long-term benefit of substantial dietary modifications for the maintenance of muscle mass and function. If so, strategies to improve compliance to protein intake suggestions, especially the per-meal threshold of 0.4 g/kg BW, need to be developed. Based on our observation, one simple recommendation for women could be to eat more frequently, like they do on Sundays.

## Figures and Tables

**Table 1:** Characteristics of healthy community-dwelling older adults (median [IQR]).

	All	Women	Men	*p* *
	*n* = 140	*n* = 72	*n* = 68	
Age [years]	77.0 [76.0–80.0]	77.0 [76.0–80.0]	78.0 [76.0–80.0]	0.559
GDS [points]	1.0 [0.0–2.0]	1.0 [0.0–2.0]	1.0 [0.0–1.8]	0.809
MMSE [points]	29.0 [29.0–30.0]	29.0 [29.0–30.0]	29.0 [29.0–30.0]	0.799
MNA [points]	27.5 [25.6–28.5]	27.5 [25.5–28.5]	27.5 [26.0–28.5]	0.666
SPPB [points]	12.0 [11.0–12.0]	12.0 [11.0–12.0]	12.0 [11.0–12.0]	0.223
BMI [kg/m^2^] ^a^	26.7 [23.5–29.2]	25.7 [23.0–28.7]	27.6 [24.7–29.3]	0.074
Body weight [kg] ^a^	74.0 [63.5–82.9]	65.8 [59.8–75.4]	81.6 [72.4–89.2]	<0.001
Fat free mass [kg]	46.1 [38.4–55.6]	38.6 [34.1–41.5]	55.9 [51.6–59.9]	<0.001
Skeletal muscle mass [kg]	19.8 [15.6–26.1]	15.7 [14.2–17.2]	26.2 [24.7–28.2]	<0.001
Skeletal muscle index [kg/m^2^]	7.3 [6.0–8.8]	6.1 [5.6–6.6]	8.8 [8.3–9.2]	<0.001
Living alone [%]	55.7	73.6	36.8	<0.001
Main meal usually alone [%]	55.7	70.8	39.7	<0.001
Appetite very good/good [%]	87.9	84.7	91.2	0.243
Energy intake [kcal]	1892 [1598–2184]	1678 [1524–1928]	2111 [1820–2327]	<0.001
Carbohydrate intake [g/day]	190.4 [161.4–236.9]	175.2 [153.8–211.2]	211.6 [177.8–257.4]	<0.001
Fat intake [g/day]	78.5 [68.2–95.0]	74.4 [67.6–89.3]	86.7 [73.7–97.5]	0.008
Protein intake [g/day]	68.1 [57.0–81.9]	63.0 [53.1–73.0]	73.6 [63.2–89.8]	<0.001
Alcohol intake [g/day]	6.6 [1.2–16.9]	3.5 [0.7–8.2]	11.5 [3.5–27.1]	<0.001

^a^ Normally distributed, * Mann-Whitney U test, *t*-test or Chi² test. Abbreviations: IQR: interquartile range; GDS: Geriatric Depression Scale (score range: 0–15); MMSE: Mini Mental State Examination (score range: 0–30); MNA: Mini Nutritional Assessment (score range: 0–30); SPPB: Short Physical performance Battery (score range: 0–12).

## References

[1] Deutz N.E., Bauer J.M., Barazzoni R., Biolo G., Boirie Y., Bosy-Westphal A., Cederholm T., Cruz-Jentoft A., Krznaric Z., Nair K.S. (2014). Protein intake and exercise for optimal muscle function with aging: Recommendations from the ESPEN Expert Group. Clin. Nutr..

[2] Bauer J., Biolo G., Cederholm T., Cesari M., Cruz-Jentoft A.J., Morley J.E., Phillips S., Sieber C., Stehle P., Teta D. (2013). Evidence-based recommendations for optimal dietary protein intake in older people: A position paper from the PROT-AGE Study Group. J. Am. Med. Dir. Assoc..

[3] Paddon-Jones D., Campbell W.W., Jacques P.F., Kritchevsky S.B., Moore L.L., Rodriguez N.R., van Loon L.J. (2015). Protein and healthy aging. Am. J. Clin. Nutr..

[4] Murphy C.H., Oikawa S.Y., Phillips S.M. (2016). Dietary Protein to Maintain Muscle Mass in Aging: A Case for Per-meal Protein Recommendations. J. Frailty Aging.

[5] Breen L., Phillips S.M. (2011). Skeletal muscle protein metabolism in the elderly: Interventions to counteract the ‘anabolic resistance’ of ageing. Nutr. Metab..

[6] Gorissen S.H.M., Witard O.C. (2017). Characterising the muscle anabolic potential of dairy, meat and plant-based protein sources in older adults. Proc. Nutr. Soc..

[7] Haines P.S., Hama M.Y., Guilkey D.K., Popkin B.M. (2003). Weekend eating in the United States is linked with greater energy, fat, and alcohol intake. Obes. Res..

[8] De Castro J.M. (2002). Age-related changes in the social, psychological, and temporal influences on food intake in free-living, healthy, adult humans. J. Gerontol. A Biol. Sci. Med. Sci..

[9] Yang P.H., Black J.L., Barr S.I., Vatanparast H. (2014). Examining differences in nutrient intake and dietary quality on weekdays versus weekend days in Canada. Appl. Physiol. Nutr. Metab..

[10] McCarthy S. (2014). Weekly patterns, diet quality and energy balance. Physiol. Behav..

[11] An R. (2016). Weekend-weekday differences in diet among U.S. adults, 2003–2012. Ann. Epidemiol..

[12] Orsama A.L., Mattila E., Ermes M., van Gils M., Wansink B., Korhonen I. (2014). Weight rhythms: Weight increases during weekends and decreases during weekdays. Obes. Facts.

[13] Farsijani S., Morais J.A., Payette H., Gaudreau P., Shatenstein B., Gray-Donald K., Chevalier S. (2016). Relation between mealtime distribution of protein intake and lean mass loss in free-living older adults of the NuAge study. Am. J. Clin. Nutr..

[14] Farsijani S., Payette H., Morais J.A., Shatenstein B., Gaudreau P., Chevalier S. (2017). Even mealtime distribution of protein intake is associated with greater muscle strength, but not with 3-y physical function decline, in free-living older adults: The Quebec longitudinal study on Nutrition as a Determinant of Successful Aging (NuAge study). Am. J. Clin. Nutr..

[15] Lord C., Chaput J.P., Aubertin-Leheudre M., Labonte M., Dionne I.J. (2007). Dietary animal protein intake: Association with muscle mass index in older women. J. Nutr. Health Aging.

[16] Kobayashi S., Asakura K., Suga H., Sasaki S. (2013). High protein intake is associated with low prevalence of frailty among old Japanese women: A multicenter cross-sectional study. Nutr. J..

[17] Loenneke J.P., Loprinzi P.D., Murphy C.H., Phillips S.M. (2016). Per meal dose and frequency of protein consumption is associated with lean mass and muscle performance. Clin. Nutr..

[18] Mishra S., Goldman J.D., Sahyoun N.R., Moshfegh A.J. (2018). Association between dietary protein intake and grip strength among adults aged 51 years and over: What We Eat in America, National Health and Nutrition Examination Survey 2011–2014. PLoS ONE.

[19] Rahi B., Colombet Z., Gonzalez-Colaco Harmand M., Dartigues J.F., Boirie Y., Letenneur L., Feart C. (2016). Higher Protein but Not Energy Intake Is Associated with a Lower Prevalence of Frailty Among Community-Dwelling Older Adults in the French Three-City Cohort. J. Am. Med. Dir. Assoc..

[20] Granic A., Mendonca N., Sayer A.A., Hill T.R., Davies K., Adamson A., Siervo M., Mathers J.C., Jagger C. (2017). Low protein intake, muscle strength and physical performance in the very old: The Newcastle 85+ Study. Clin. Nutr..

[21] Isanejad M., Mursu J., Sirola J., Kroger H., Rikkonen T., Tuppurainen M., Erkkila A.T. (2016). Dietary protein intake is associated with better physical function and muscle strength among elderly women. Br. J. Nutr..

[22] Gingrich A., Spiegel A., Kob R., Schoene D., Skurk T., Hauner H., Sieber C.C., Volkert D., Kiesswetter E. (2017). Amount, Distribution, and Quality of Protein Intake Are Not Associated with Muscle Mass, Strength, and Power in Healthy Older Adults without Functional Limitations-An enable Study. Nutrients.

[23] D’Ath P., Katona P., Mullan E., Evans S., Katona C. (1994). Screening, detection and management of depression in elderly primary care attenders. I: The acceptability and performance of the 15 item Geriatric Depression Scale (GDS15) and the development of short versions. Fam. Pract..

[24] Cwikel J., Ritchie K. (1989). Screening for depression among the elderly in Israel: An assessment of the Short Geriatric Depression Scale (S-GDS). Isr. J. Med. Sci..

[25] Folstein M.F., Robins L.N., Helzer J.E. (1983). The Mini-Mental State Examination. Arch. Gen. Psychiatry.

[26] Guigoz Y., Vellas B., Garry P.J. (1996). Assessing the nutritional status of the elderly: The Mini Nutritional Assessment as part of the geriatric evaluation. Nutr. Rev..

[27] Guralnik J.M., Ferrucci L., Pieper C.F., Leveille S.G., Markides K.S., Ostir G.V., Studenski S., Berkman L.F., Wallace R.B. (2000). Lower extremity function and subsequent disability: Consistency across studies, predictive models, and value of gait speed alone compared with the short physical performance battery. J. Gerontol. A Biol. Sci. Med. Sci..

[28] Janssen I., Heymsfield S.B., Baumgartner R.N., Ross R. (2000). Estimation of skeletal muscle mass by bioelectrical impedance analysis. J Appl. Physiol..

[29] Dehne L.I., Klemm C., Henseler G., Hermann-Kunz E. (1999). The German Food Code and Nutrient Data Base (BLS II.2). Eur. J. Epidemiol..

[30] Bollwein J., Diekmann R., Kaiser M.J., Bauer J.M., Uter W., Sieber C.C., Volkert D. (2013). Distribution but not amount of protein intake is associated with frailty: A cross-sectional investigation in the region of Nurnberg. Nutr. J..

[31] Beasley J.M., Wertheim B.C., LaCroix A.Z., Prentice R.L., Neuhouser M.L., Tinker L.F., Kritchevsky S., Shikany J.M., Eaton C., Chen Z. (2013). Biomarker-calibrated protein intake and physical function in the Women’s Health Initiative. J. Am. Geriatr. Soc..

[32] Deutsche Gesellschaft für Ernährung: Wie viel Protein Brauchen wir?. https://www.dge.de/wissenschaft/referenzwerte/protein/.

[33] Cardon-Thomas D.K., Riviere T., Tieges Z., Greig C.A. (2017). Dietary Protein in Older Adults: Adequate Daily Intake but Potential for Improved Distribution. Nutrients.

[34] Gorissen S.H., Horstman A.M., Franssen R., Kouw I.W., Wall B.T., Burd N.A., de Groot L.C., van Loon L.J. (2017). Habituation to low or high protein intake does not modulate basal or postprandial muscle protein synthesis rates: A randomized trial. Am. J. Clin. Nutr..

[35] McNamara D.J. (2015). The Fifty Year Rehabilitation of the Egg. Nutrients.

[36] Robinson S.M., Jameson K.A., Batelaan S.F., Martin H.J., Syddall H.E., Dennison E.M., Cooper C., Sayer A.A. (2008). Diet and its relationship with grip strength in community-dwelling older men and women: The Hertfordshire cohort study. J. Am. Geriatr. Soc..

[37] Tieland M., Borgonjen-Van den Berg K.J., Van Loon L.J., de Groot L.C. (2015). Dietary Protein Intake in Dutch Elderly People: A Focus on Protein Sources. Nutrients.

[38] Rousset S., Patureau Mirand P., Brandolini M., Martin J.F., Boirie Y. (2003). Daily protein intakes and eating patterns in young and elderly French. Br. J. Nutr..

[39] Livingstone M.B., Black A.E. (2003). Markers of the validity of reported energy intake. J. Nutr..

